# Adaptation of *Salmonella enterica *Hadar under static magnetic field: effects on outer membrane protein pattern

**DOI:** 10.1186/1477-5956-10-6

**Published:** 2012-02-03

**Authors:** Sarra Snoussi, Alya El May, Laurent Coquet, Philippe Chan, Thierry Jouenne, Ahmed Landoulsi, Emmanuelle DÉ

**Affiliations:** 1 UMR 6270 CNRS, Faculté des Sciences, Université de Rouen, 76821, Mont Saint Aignan Cedex, France; 2 Laboratoire de Biochimie et Biologie Moléculaire, Faculté des Sciences de Bizerte, Université de Carthage, Zarzouna, Bizerte, Tunisie

**Keywords:** *Salmonella*, Static magnetic field, Outer membrane proteome

## Abstract

**Background:**

*Salmonella enterica *serovar Hadar (*S*. Hadar) is a highly prevalent foodborne pathogen and therefore a major cause of human gastroenteritis worldwide. Outer membrane proteins whose production is often regulated by environmental conditions also play important roles in the adaptability of bacterial pathogens to various environments.

**Results:**

The present study investigated the adaptation of *S*. Hadar under the effect of acute static magnetic field exposure (200 mT, 9 h) and the impact on the outer membrane protein pattern. *Via *two-dimensional electrophoresis (2-DE) and LC-MS/MS spectrometry, we compared the proteome of enriched-outer membrane fraction before and after exposure to a magnetic field. A total of 11 proteins, displaying more than a two-fold change, were differentially expressed in exposed cells, among which 7 were up-regulated and 4 down-regulated. These proteins were involved in the integrity of cell envelope (TolB, Pal), in the response to oxidative stress (OmpW, dihydrolipoamide dehydrogenase, UspF), in the oxidative stress status (bacterioferritin), in virulence (OmpX, Yfgl) or in motility (FlgE and UspF). Complementary experiments associated the down-regulation of FlgE and UspF with an alteration of swarming, a flagella-driven motility, under SMF. Furthermore, the antibiotic disc diffusion method confirmed a decrease of gentamicin susceptibility in exposed cells. This decrease could be partly associated with the up-regulation of TolC, outer membrane component of an efflux pump. OmpA, a multifunctional protein, was up-regulated.

**Conclusions:**

SMF (200 mT) seems to maintain the cell envelope integrity and to submit the exposed cells to an oxidative stress. Some alterations suggest an increase of the ability of exposed cells to form biofilms.

## Background

A large number of attempts to explain biological effects of magnetic fields at the molecular level have been reported for prokaryotes and eukaryotes [1-4]. Usually, biological materials that are used for such investigations are cells [5-7], tissues [8], and living organisms [1,9]. Viability and proliferation [10,11], activity of enzymes [1], transport of ions [12] and gene transcription [13] are the common fields of investigation. All these studies gave contradictory results. Thus, it has been reported that magnetic field (MF) treatment (10 mT, 50 Hz) on different strains of *Escherichia coli, Leclercia adecarboxylata*, and *Staphylococcus aureus *have induced cell mortality, which was time exposure and/or MF intensity, and strain-dependent [10]. At the opposite, Tsuchiya et al. (1999) [14] reported that high MFs (ranging from 5.2 to 6.1 T) were less detrimental. *E. coli *cells exposed to an extremely low-frequency magnetic field (0.1 T) for 6.5 h exhibited 100 times higher viability as compared to unexposed cells [15]. Nascimento et al. (2003) [16] demonstrated that an increase of glucose transport into *E. coli *cells was involved in the bacterial growth exacerbation after 8 h of exposure to an electromagnetic field (0.5 mT, 60 Hz). Consequently, it appears that the MF effects on bacterial growth and viability are dependent on the applied conditions/parameters as well as on the strain used.

In other respects, several studies revealed the impact of MF on gene expression. Magnetic field (1.10 mT, 60 Hz) could stimulate the σ^32 ^expression, a stress promoter transcription factor in *E. coli *[17] , or enhances the transcription of the *rpoS *gene in *E. coli *when being inhomogeneous (ranging from 5.2 to 6.1 T) [14]. It could also stimulate transposition activity mediated by the synthesis and accumulation of the heat-shock proteins DnaK/J (when it is set at 1.2 mT, 50 Hz) [18].

*Salmonella *spp. is a leading cause of bacterial foodborne disease all over the world, causing a diversity of illnesses including typhoid fever, gastroenteritis and septicemia. As a foodborne pathogen, it is a good working prokaryotic model for studying SMF impact. We have previously described the effect of a static magnetic field (SMF, 200 mT) in *S*. Hadar. SMF induced a decrease of the cell viability when applied between 3 and 6 h whereas the growth re-increased after 6 to 9 h of exposure. The analysis of the differential expression of genes under SMF exposure showed that the expression level of the 16S-rRNA mRNA remained stable allowing its use as a reference gene. Interestingly, mRNAs of *rpoA*, *katN*, and *dnaK *genes were over-expressed after 10 h of exposure. This suggests a possible stress response and adaptation of *S*. Hadar to SFM [11]. A more recent study showed that homogenous SMF(159.2 mT), applied for up to 24 h on different bacteria species spread on agar plates, failed to affect their viability [19]. These contradictory results could be explained by the differences in culture mode and/or medium composition [7], the direction and the homogeneity of the magnetic field reaching the bacteria growing on an agar plate or in a liquid medium. Indeed, it was shown for *E. coli *that the adherence to surfaces is dependent on the type of surface and direction of the magnetic induction towards the surface colonized by the cells (data not published). Many membrane proteins, especially transporters, play a crucial role in adaptation of bacteria to environmental stress [20] and in their resistance to antibiotics [21]. However, no study has yet been performed to analyze the changes inflicted on membrane proteins by SMF and their impact on the bacteria physiology. In this context, the objectives of this study were to investigate the modifications of *S*. Hadar outer membrane protein (OMP) patterns induced by a SMF stress (200 mT, 9 h). Our proteomic investigations showed that SMF exposure may alter some cellular functions, e.g., the integrity of the cell envelope, the bacterial virulence/motility and modulate the response to oxidative stress.

## Results

### Comparison of enriched-outer membrane protein patterns after exposure to SMF

**Figure 1:**
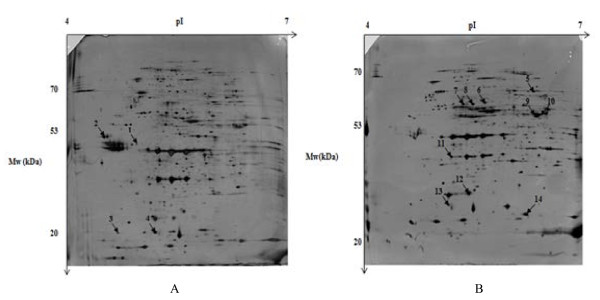
**2-DE gels of enriched-OMP fractions of *Salmonella *Hadar**. **A**: with no SMF exposure. Arrows and numbers point the proteins differentially expressed that are mentioned in Table 1, **B**: after exposure to SMF (200 mT for 9 h). One hundred μg of proteins have been loaded. Proteins were stained by silver nitrate.

In order to evaluate the impact of SMF on enriched outer membrane (OM) protein fraction of S. Hadar, we compared the proteomes of enriched-OMP fractions from non-exposed and 200 mT SMF exposed cells. Typical 2-DE gels obtained from exposed and non-exposed organisms are shown in Figure 1. A total of 14 spots exhibited a change in their amount following SMF treatment. Their location on the 2-DE gel is shown in Figure 1A. These proteins were then identified by LC-MS/MS (Table 1) and corresponded to 11 different polypeptides. Thus, in the exposed cells, four proteins were down-regulated, i.e., the flagellar hook protein (FlgE), the outer membrane protein assembly complex subunit YfgL (YfgL), the outer membrane protein × (OmpX) and bacterioferritin (Bfr), whereas 7 proteins were up-regulated, i.e., the dihydrolipoamide dehydrogenase (Lpd), the outer membrane channel TolC (three TolC isoforms and a TolC precursor), the translocation protein TolB, the outer membrane protein A (OmpA), the outer membrane protein W (OmpW), the peptidoglycan-associated outer membrane lipoprotein (Pal) and the hypothetical protein STY1416.

**Table 1:** Identification of differentially expressed outer membrane proteins of *Salmonella enterica *Hadar SH287 exposed to magnetic field (200 mT for 9 h).

Spot n°	fold change	M(Da)	pI	Peptides Matched (score > 51)	Mascot score	Coverage (%)	Identification (accession n°.)	Gene	Function and location
**Downregulated proteins**									

**1**	2.3	41988	4.42	3	198	9	flagellar hook protein FlgE (ss1980)	*flgE*	Ciliary or flagellar motility
**2**	4.2	41888	4.65	2	67	5	OM protein assembly complex subunit YfgL (ss2008)	*yfgL*	Homeostatic control
**3**	2.6	18483	5.74	8	399	47	outer membrane protein × (ss3443)	*ompX*	Adhesion and resistance
**4**	3.6	18370	4.64	3	150	24	bacterioferritin (ss3466)	*bfr*	Iron detoxification

**Upregulated proteins**									

**5**	2.9	45659	6.12	67	1514	68	dihydrolipoamide dehydrogenase (ss1304)	*dld*	Oxidoreductase
**6**	1.9	53653	5.42	6	229	11	outer membrane channel (ss1640)	*tolC*	Protein transport
**7**	1.5	53653	5.42	5	202	10	outer membrane channel (ss1645)	*tolC*	Protein transport
**8**	2	53637	5.43	7	402	16	outer membrane protein TolC precursor tolC (ss 1649)	*tolC*	Protein transport
**9**	1.5	53693	5.42	2	149	6	RecName: Full = Outer membrane protein tolC (ss 1881)	*tolC*	Protein transport
**10**	2.2	46119	7.85	16	611	38	translocation protein TolB (ss1775)	*tolB*	Protein import
**11**	2.2	37492	5.60	22	752	56	outer membrane protein A ss 2655	*ompA*	Porin
**12**	2.2	22978	5.64	9	268	24	outer membrane protein W (ss 3270)	*ompW*	Bacterial porin
**13**	3.1	18853	6.29	13	336	57	peptidoglycan- associated outer membrane lipoprotein(ss 3407)	*pal*	Integrity of cell envelope
**14**	2.3	15704	5.93	9	351	44	hypothetical protein STY1416 (ss 3598)	*sty1416*	Unknown function

Differential expression is shown as fold change (minimum 2-fold, *P*-value < 0.05)

### Effects of SMF on antibiotic susceptibility

In order to determine the biological effects of SMF on *S*. Hadar antibiotic susceptibility, a standardized bioassay using the disc diffusion method was applied. The diameter of inhibition zones was determined for non-exposed and exposed cells.

**Figure 2:**
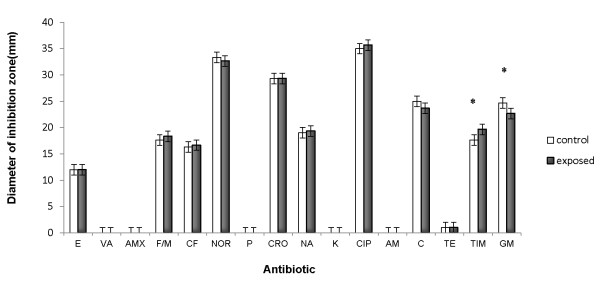
**Effect of SMF (200 mT, 9 h) exposure on susceptibility of *Salmonella *Hadar to antibiotics**. Comparison of the diameter of the inhibition zones of different antibiotics: penicillin (P), cephalotin (CF), tetracycline (TE), erythromycin (E), chloramphenicol (C), nalidixic acid (NA), vancomycin (VA), amoxicillin (AMX), nitrofurantoin (F/M), norfloxacin (NOR), ceftriaxone (CRO), kanamycin (K), ciprofloxacin (CIP), ampicillin (AM), gentamicin (G) and ticarcillin + clavulanic acid (TIM). The values are the means ± SD (n = 3, Mann-Whitney *U *test).

The inhibition zone diameters obtained for the different tested antibiotics are given on Figure 2. Results pointed out a significant (*p *< 0.05) decrease of the bacterial susceptibility only for gentamicin. In contrast, data showed a significant increase (*p *< 0.05) of the ticarcillin + clavulanic acid mixture efficiency. No significant effect of SMF exposure was observed for penicillin, cephalotin, tetracycline, erythromycin, chloramphenicol, nalidixic acid, vancomycin, amoxicillin, nitrofurantoin, norfloxacin, ceftriaxon, kanamycin, ciprofloxacin and ampicillin sensitivity.

### Effects of SMF on bacterial motility

**Figure 3:**
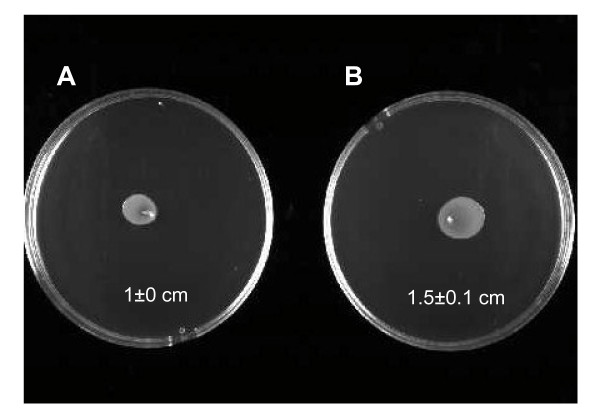
**Motility assays of *Salmonella enterica *Hadar after exposure to SMF (200 mT, 9 h)**. Swarming test: Cells were inoculated with a needle into the bottom of Nutrient broth + agar (0.6%) medium. Plates were incubated at 37°C for 24 h. The values are the means ± SD (n = 3, Mann-Whitney *U *test). A: Exposed; B: Non-exposed.

Due to the down-regulation of FlgE by exposed cells, we investigated the effect of SMF on bacterial motility, i.e., swarming and twitching. The swarming (a flagella-driven movement) was altered. As shown by the Figure 3 the zone of motility for the exposed cells is 1 ± 0 cm and 1.5 ± 0.1 cm for the non-exposed (*p *< 0.05). SMF had no effect (*p *> 0.05) on the twitching, a type IV pili-dependent motility (data not shown).

## Discussion

Outer membrane proteins are essential for maintaining the integrity and selective permeability of bacterial membranes [22]. They also play an important role in antibiotic resistance [21]. Due to its cellular location, OM is highly sensitive to environmental conditions prevailing in the extra-cellular medium. This is important for bacterial pathogenesis because it enhances the adaptability of pathogens to various environments [20,23]. We have previously reported a SMF effect on cell viability and gene expression of *S*. Hadar [11]; we now address the question of the SMF impact on the OMPs pattern of this pathogen.

Impact of SMF on cell envelope integrity. The peptidoglycan-associated lipoprotein (Pal) and the TolB protein were found to be up-regulated after SMF exposure. TolB, a periplasmic protein, is partially associated with the outer membrane through a specific interaction with Pal [24]. The Tol/Pal system of *Escherichia coli *is composed of the YbgC, TolQ, TolA, TolR, TolB, Pal and YbgF proteins. It is involved in maintaining the integrity of the outer membrane [25]. Two possible functions have emerged for the Tol/Pal system: its involvement in cell envelope biogenesis and/or as a tether in cell division that maintains the appropriate juxtaposition of the two membranes relative to the peptidoglycan layer [26]. It could be hypothesized that the SMF increased the expression of the system involving the TolB and the peptidoglycan-associated lipoprotein Pal in order to maintain the envelope integrity and thus cell division. This is confirmed by a renewal of the cell viability observed following 9 h of SMF exposure [11]. These data suggest a contribution of TolB and Pal proteins in mechanisms of defense in order to overcome the stress effects of the SMF.

Proteins involved in oxidative stress. Three overexpressed proteins may be related to the oxidative stress response. The OmpW protein belongs to a family of small OMPs involved in the transport of small hydrophobic molecules across the outer membrane [27]. It also may be involved in the protection of bacteria against various forms of environmental stress. Recent studies have indicated that its expression is activated in response to oxidative stress [28]. The up-regulated dihydrolipoamide dehydrogenase (Lpd) is a component of the pyruvate dehydrogenase complex (PDC) that connects glycolysis and tricarboxylic acid cycle enzymes. PDC carries out the conversion of pyruvate to acetyl-CoA. Haramaki et al. (1997) [29] suggested an antioxidant function for Lpd, related to the role of free lipoic acid as an antioxidant. The up-regulated STY1416 protein, identified as the universal stress protein UspF, has been described to confer resistance against oxidative stress. It has, however, a minor role compared to other members of the Usp family [30]. Therefore, as shown in eukaryotes [2-4,31], SMF exposure might induce an oxidative stress in *Salmonella*. The down-regulation of bacterioferritin in cells under magnetic field exposure may lead to a decrease of iron storage and thus to the impairment of iron detoxification. This observation suggests a contribution of the bacterioferritin down-regulation in the oxidative stress status, a correlation being established between iron homeostasis involving bacterioferritin and the oxidative stress response [32-34].

Impact of SMF on the *Salmonella *virulence. Two proteins involved in virulence, OmpX and YfgL, were down-regulated under SMF. The integral OmpX belongs to a family of virulence-related membrane proteins which promotes mammalian cell adhesion and invasion and helps to defend against the human complement system [35]. This small channel could also play a role in the antibiotic resistance as it is up-regulated in conditions where the Omp35 and Omp36 major porins in *Enterobacter aerogenes *have a decreased expression [36]. However, we did not detect an increasing expression of the *Salmonella *major porins, as we could expect concomitantly to the under-expression of OmpX. In other respects, the outer membrane lipoprotein YfgL is involved in virulence of *Salmonella *as it plays a crucial role in the regulation of the expression of all *Salmonella *TTSS systems (Type Three Secretion Systems) that occurs during host infection. Indeed, three TTSS (TTSS-1, TTSS-2, and flagella) allow the bacterium to cross the intestinal barrier and to disseminate systemically [37]. The deletion of *yfgL *gene in *S. enterica *serovar Enteritidis led to the transcriptional down-regulation of the flagellar genes encoding the TTSS structural proteins and of the effector proteins secreted by these TTSS [37]. This down-expression of both ompX and YfgL suggests a possible impairment of the virulence under a SMF exposure (200 mT, 9 h).

Impact of SMF on the *Salmonella *motility. For a lot of pathogens, virulence and motility are often intimately linked by complex regulatory networks [38]. Thus, we observed a down-regulation of the hook flagellar protein FlgE by exposed cells. In accordance with this result, SMF-exposed cells exhibited a decrease in the swarming motility, a flagella-driven movement. The flagellar hook, a constituent of the bacterial motile flagellum, is a short connection between the flagellar motor and the long filament acting as a helical propeller. It is made of about 120 copies of a single protein, FlgE, and its function is essential for dynamic and efficient bacterial motility and taxis [39]. Confirming this bacterial motility decrease was the up-regulation of the STY1416 protein, which was shown to exhibit a negative effect on bacterial motility [30].

Effects of SMF on antibiotic susceptibility. As pointed out by proteomic data, SMF exposure induced an overexpression of the TolC protein. This protein is the outer membrane component of the efflux pump AcrAB-TolC, an efflux system known to play an important role in the multidrug resistance in *Salmonella *[40]. TolC is required for the functioning of seven drug efflux systems in *S. enterica *serovar Typhimurium [41]. In this context, we compared the susceptibility of *S*. Hadar to different antibiotics before and after SMF exposure. Our data showed no influence of SMF on antibiotic susceptibility, except for gentamicin that underwent a significant decrease of its effectiveness and for ticarcillin + clavulanic acid mixture that acts in an unexpected increasing manner (Figure 2). Efflux pumps have already been described as involved in the resistance to aminoglycoside in *Pseudomonas aeruginosa *[42]. These pumps usually play an essential role in export of different structurally unrelated substrates ranging from antimicrobials, heavy metals, and detergents to large toxins [43]. If we cannot exclude a role of TolC accumulation in the decrease of susceptibility of exposed cells to gentamicin, it was actually not sufficient to promote an effect on other antibiotic susceptibility. We can consequently imagine that the decrease of susceptibility to gentamicin by SMF exposure might also result from other mechanisms. Indeed, gentamicin is a cationic antibiotic which binds reversibly to anionic sites of the bacterial cell membrane dependent on concentration [44]. Its binding to anionic phospholipids (such as cardiolipids) of the OM facilitates the rupture of this membrane [45]. The membrane lipid composition and modification, especially cardiolipid's proportion, should therefore be examined after SMF exposure.

Electromagnetic fields have been hypothesized to affect the membrane permeability through modifications on channel-forming proteins [46]. Since we did not detect any differential expression of the OmpC and OmpF porins, we could hypothesize that the SMF may alter kinetics of beta-lactamase inhibition by clavulanic acid, thus reducing beta-lactamase activity.

Accumulation of major outer membrane protein OmpA. We observed the up-regulation of OmpA by SMF exposed cells. This OMP is a multifaceted protein which can function as an adhesin and invasin, participate in biofilm formation, act as both an immune target and evasin, and serve as a receptor for several bacteriophages [47]. The structure of OmpA has given many insights into the possible porous nature of this protein. However, the definition of molecules that utilize this channel into the cell has not been resolved [47]. OmpA is the predominant cell surface antigen in *Enterobacteria*, found in about 100,000 copies per cell [47]. Furthermore, OmpA-sal modulates the adaptive immune responses to *S. enterica *serovar Typhimurium by activating dendritic cells and initiates an adaptive immune response, two important properties to be considered in the development of effective *S. enterica *serovar Typhimurium vaccines [48]. OmpA expression is controlled by two stress-responsive ribonucleolytic mechanisms, and the environmental stimuli regulating OmpA expression could be transduced through these pathways in response to membrane stress [47]. Concomitantly to the disappearance of the flagellum, the overexpression of this adhesion involved in the first steps of biofilm formation raises questions about a possible enhancement of the adhesion capacities of the bacterium.

## Conclusions

Using a proteomic approach we obtained an overview of *S*. Hadar OMPs, which were differentially expressed under SMF stress. These proteins were involved in the integrity of cell envelope (TolB, Pal), in the response to oxidative stress (OmpW, dihydrolipoamide dehydrogenase, UspF), in the oxidative stress status (bacterioferritin), in virulence (OmpX, Yfgl) or in motility (FlgE and UspF). Complementary experiments associated the down-regulation of FlgE and UspF with an alteration of swarming motility under SMF. Furthermore, we showed a decrease of gentamicin susceptibility, associated with the up-regulation of TolC. This study enhances knowledge of the biological effects of the SMF. Whereas the cell envelope integrity seems to be maintained, exposed cells are submitted to an oxidative stress. Some alterations suggest an increase of the ability of exposed cells to form biofilms. Experiments aiming to confirm this hypothesis are currently in progress.

## Methods

### Bacterial growth and magnetic field exposure

The bacterium *Salmonella enterica *subsp. enterica serovar Hadar (isolate 287) (6, 8: Z10: e, n, X) was provided by the Institut Pasteur de Tunis (Tunisia) and stored at -80°C. Nutrient broth (Pronadisa, Hispanlab, Madrid, Spain) and nutrient agar (15 g/L) were used for cultivation of bacteria.

Precultures were performed overnight at 37°C in 10 mL of culture medium in 18-mm diameter tubes and then diluted to the same initial concentrations corresponding to 0.1 OD600 in 70 ml in an Erlenmeyer-shaped glass double phial (external diameter, 6 cm; external height, 8 cm; internal diameter, 4 cm; internal height, 7 cm). The SMF was generated by a Helmholtz coil, i.e., two bobbins (diameter 20 cm, length 13 cm, each), powered by a transformer. The two bobbins were separated by 6 cm. Magnetic induction was perpendicular to the double glass phial and thus to the bacterial suspension. The induction of SMF was measured and standardized using a Teslameter (CA 42, Chauvin Arnoux). The bobbins were water-cooled, and the temperature inside was regulated at the value of the laboratory temperature (≈ 25°C). The temperature was maintained at 37°C inside the glass double phial by water circulation, using an incubator system composed of pump and resistance. The samples were positioned in the center between the two bobbins. The SMF induction was 200 ± 8 mT. The background SMF value was 60 ± 5 μT.

Bacterial cultures were exposed to magnetic field during 9 h; subsequently, the culture was centrifuged at 2000 g for 10 min. For the control experiments, the bacterial cultures were similarly positioned, except that the magnetic field was turned off.

### Outer membrane protein extract preparation

Enriched-outer-membrane extracts were prepared from bacterial pellets using ultracentrifugation and a sarkosyl extraction method [49,50]. Briefly, free cells were harvested for 15 min at 2000 g, the pellet was then resuspended in 20 mM Tris-HCl and 0,7% lysozyme. The suspension was sonicated (pulses 1 s on/1 s off, time 1 min, amplification 25%, Vibra Cell 75115, Bioblock Scientific, Illkirch, France), incubated for 30 min at 37°C and then centrifuged 10 min at 10,000 g to separate the supernatant (S) from the cellular debris (C).

Membrane proteins (C_0_) were pelleted at 60,000 g for 45 min at 20°C (Beckman Coulter TL100 ultracentrifuge) and the supernatant (S_0_) was considered as soluble proteins. The pellet C_0 _was suspended in 4 ml (for 1 L of culture) of Tris-HCl 10 mM and 0.3% sarkosyl for 30 min at room temperature. An ultracentrifugation (60,000 g for 45 min at 4°C) allows the separation of an enriched-inner membrane protein fraction in the supernatant (S_1_) and enriched-outer membrane protein fraction in the pellet (C_1_). Pellet was re-suspended in 2 ml of Tris- HCl 10 mM, assayed (Bio-Rad protein assay, Biorad, France) and stored at -20°C.

### Two-dimensional gel electrophoresis

For the first dimension (IEF), 100 μg of proteins were solubilized in 400 μL IEF buffer composed of 7 M urea, 2 M thiourea, 0.1% (w/v) ASB, 14, 2 mM tributyl phosphine and 0.4% (w/v) Coomassie blue. The first-dimension separation was carried out with immobilized pH gradients (Immobiline DryStrip pH 4-7 non-linear, 18 cm; Amersham Pharmacia Biotech). IEF was performed within the IEF cell (Bio-Rad) as follows: active rehydratation for 12 h at 50 V, 250 V for 15 min, gradient from 250 V to 10,000 V for 3 h and final focusing for 12 h at 10,000 V. Strips containing focused proteins were then stored at -20°C. After the IEF step, the strip was equilibrated in a buffer containing 1% dithiothreitol for 10 min. A second equilibration step was performed for 10 min in equilibration buffer containing 4% iodoacetamide. The second dimension of separation was ensured by sodium dodecyl sulfate polyacrylamide gel electrophoresis using 12.5% polyacrylamide resolving gel (width 16 cm, length 20 cm, thickness 0.75 cm). Experiments were carried out using the protean II Xi vertical systems (Biorad). Proteins were visualized after silver staining. Images of gels were acquired using the ProXPRESS Proteomic Imaging System (Perkin Elmer) with a resolution of 100 μm.

### Protein identification

Protein spots were manually excised from 2-D gels. Excised spots were washed three times with water, once with acetonitrile (CH_3_CN) and dried for 2 h. Trypsin digestion was performed overnight with a dedicated automated system (MultiPROBE II, PerkinElmer). The gel fragments were subsequently incubated twice for 15 min in a H_2_O/CH_3_CN solution, once for 15 min in 1% (v/v) Formic Acid and once with 100% ACN to allow extraction of peptides from the gel pieces. Peptide extracts were then dried and dissolved in starting buffer for chromatographic elution, consisting of 3% CH_3_CN and 0.1% HCOOH in water. Peptides were enriched and separated using a nano-LC1200 system coupled to a 6340 Ion Trap mass spectrometer equipped with a HPLC-chip cube interface (Agilent Technologies, Massy, France). The fragmentation data were interpreted using the Data Analysis program (version 3.4, Bruker Daltonic, Billerica, MA, USA). For protein identification, MS/MS peak lists were extracted, converted into mgf-format files and compared with the *Salmonella *protein database using the MASCOT Daemon (version 2.1.3; Matrix Science, London, UK) search engine. The searches were performed with variable modifications for oxidation of methionines, carbamidomethylation and carboxymethylation and with a maximum of one missed cleavage. MS/MS spectra were searched with a mass tolerance of 1.6 Da for precursor ions and 0.8 for MS/MS fragments. Only peptides matching an individual ion score > 51 were considered. Proteins with two or more unique peptides matching the protein sequence were automatically considered as a positive identification.

### Gel analysis

Scanned gel images (ProXpress; PerkinElmer) were imported into the image analysis software Progenesis SameSpots v3.0 (Nonlinear Dynamics). Bacterial culture and protein extraction experiments were performed 3 times per condition. For each experimental condition, two 2-D gels were matched together to form a reference image. The protein spot volumes were automatically normalized in the software. A list of spots which changed in abundance on the different gels was generated. In our study, only normalized spots exhibiting variations with a fold of at least 2 and with *p-value *(Anova) < 0.05, *q-value *(exclusion of false positive) < 0.05 and P (Power Analysis) > 0.8 were selected as being differentially expressed. Principal component analysis (PCA) was applied to analyze the similarity of protein patterns among gels and the expression profiles of protein spots fulfilling the above criteria.

### Bioinformatic tools

A prediction of unknown protein cellular location was obtained from the genome annotation of *Salmonella *(accessible at http://www.uniprot.org); (http://www.ncbi.nlm.nih.gov/).

### Antibiotic susceptibility

Antimicrobial susceptibility tests were performed using the agar diffusion method on nutrient agar. A volume of 300 μl of each bacterial suspension (3-3.6 × 10^8 ^CFU/ml) was spread on agar plates. Antibiotic disks were disposed manually. Antibiograms were performed using the following antibiotics (BioMérieux, France): penicillin 10 U (P), cephalotin 30 μg (CF), tetracycline 30 μg (TE), erythromycin 15 μg (E), chloramphenicol 30 μg (C30), nalidixic acid 30 μg (NA), vancomycin 30 μg (VA), (ticarcillin + clavulanic acid) 75 + 10 μg (TIM), amoxicillin 25 μg (AMX), nitrofurantoin 300 μg (F/M), norfloxacin 10 μg (NOR), ceftriaxone 30 μg (CRO), kanamycin 30 μg (K), ciprofloxacin 5 μg (CIP), ampicillin 10 μg (AM) and gentamicin 10 μg (GM). After incubation for 18 h at 37°C, inhibition zone diameters were measured by the standard methods.

Antibiograms were performed for exposed and non-exposed cells. For control experiments (i.e., non-exposed organisms), the bacterial cultures were similarly positioned except that MF was turned off. Experiments were performed in triplicate.

### Bacterial motility

*Twitching (type IV pili*) assays were performed with Nutrient broth (Pronadisa, Hispanlab, Madrid, Spain: 5 g polypepton and 3 g meat extract per liter of distilled water, pH 7) solidified with 1.5% agar. Twitch plates were briefly dried and strains were stab-inoculated with a sharp toothpick to the bottom of the Petri dish from an overnight-growth. After incubation at 37°C for 24 h, the zone of motility at the agar/Petri dish interface was determined [51,52].

*Swarming (flagella and type IV pili)*. Medium used for assay is a 0.6% agar Nutrient broth. Swarm plates were briefly dried and strains were stab inoculated with a sharp toothpick to the bottom of the Petri dish from an overnight-grown then. After incubation at 37°C for 24 h, the zone of motility at the agar/Petri dish interface was measured [51,52].

### Statistical analysis

The differences between control and exposed cells were determined using the nonparametric Mann-Whitney *U *test. Means were given with ± SD and the level of significance was set at *p *< 0.05.

## Abbreviations

LC-MS/MS: liquid chromatography-tandem mass spectrometry; MF: magnetic field; SMF: static magnetic field; OM: outer membrane; OMPs: outer membrane proteins; IEF: iso-electro-focalisation.

## Competing interests

The authors declare that they have no competing interests.

## Authors' contributions

SS carried out proteomic studies, analyzed data, and drafted the manuscript; AEM designed SMF studies and drafted the manuscript; LQ performed trypsin digestion and identification of the proteins, PC performed mass spectrometry experiments, TJ, AL supervised this study and ED designed OM extraction method, proteomic studies and drafted the manuscript. All authors read and approved the final manuscript.
